# Clinical and radiological results of high offset tri-lock bone preservation stem in unilateral primary total hip arthroplasty at a minimum follow-up of 3 years

**DOI:** 10.1186/s13018-021-02787-7

**Published:** 2021-10-24

**Authors:** Linbo Peng, Jun Ma, Yi Zeng, Yuangang Wu, Haibo Si, Bin Shen

**Affiliations:** Department of Orthopedics, Orthopedic Research Institute, West China Hospital, Sichuan University, 37# Guoxue Road, Chengdu, 610041 Sichuan Province People’s Republic of China

**Keywords:** Total hip arthroplasty, High offset, Stem, Hip function, Offset, Leg length discrepancy

## Abstract

**Background:**

Although the high offset Tri-Lock bone preservation stem (BPS) was used widely, few studies explored the clinical and radiological results. The purpose of this study was to determine the clinical and radiological results of high offset Tri-Lock BPS in unilateral primary total hip arthroplasty (THA) at a minimum follow-up of 3 years.

**Methods:**

55 patients who underwent cementless THA with high offset Tri-lock BPS from 2017 to 2018 were followed for a minimum follow-up of 3 years. Patients were assessed clinically for complications, Harris hip score (HHS), Western Ontario and McMaster Universities Osteoarthritis Index (WOMAC), and Oxford Hip Score (OHS). Femoral offset (FO), acetabular offset (AO), hip offset (HO), HO difference, and leg length discrepancy (LLD) were measured on the anteroposterior (AP) pelvic radiograph. Standard pelvic AP and lateral radiographs were used to evaluate for evidence of bone ingrowth, stem subsidence, stem alignment, radiolucent line around the stem, osteolysis, loosening, ectopic ossification, and femoral stress shielding.

**Results:**

No patients reported complications during hospitalization nor the follow-up period. At a mean follow-up of 42.5 months, the mean HHS, WOMAC, and OHS scores showed a significant improvement from preoperative to the latest follow-up. No patients reported thigh pain. No revision nor sign of radiographic loosening had been detected. The high offset Tri-Lock BPS significantly improved the FO and HO postoperatively. HO difference and LLD were balanced postoperatively. No sign of stem subsidence, radiolucent line, osteolysis, loosening, ectopic ossification, nor severe stress shielding (more than grade 3–4) were observed at the latest follow-up.

**Conclusion:**

The high offset Tri-Lock BPS demonstrated excellent clinical and radiographic outcomes at a minimum follow-up of 3 years. HO difference and LLD between legs decreased significantly and achieved balance postoperatively. Long-term follow-up is required for a definitive conclusion.

## Introduction

Total hip arthroplasty (THA) is an effective method to treat end-stage hip disease for relieving the pain and improving joint activity and the quality of life [1, 2]. The conventional standard-length femoral stem is a common prosthesis and associated with a high survival rate at a follow-up of nearly 30 years [3–5]. The demand for THA surgery in younger patients is increasing in recent years [6, 7]. Young patients face a higher risk of revision because of higher activity levels than elderly patients [8]. The lifespan of the cementless prosthesis is limited, and young patients may inevitably encounter revision surgery in later life [9]. The median time to revision for patients younger than 60 years was 4.4 years [10]. The conventional standard-length femoral stem may increase the difficulty of revision surgery due to osseointegration and deficiencies in the preservation of the moral bone stock [11, 12]. Besides, thigh pain, stress shielding, aseptic loosening, and periprosthetic fracture remain a matter of concern [13, 14].

Recently, shorter bone preservation stems (BPS) were widely used in THA because of specific advantages [15]. The novel design of Tri-Lock BPS, such as a reduced lateral shoulder, a thin geometry, and a shorter length, conserves native bone stock during THA [11]. Tri-Lock BPS has a highly porous and roughened coating (Gription), which leads to mechanical integrity and long-term biological fixation [16]. Furthermore, the short stem might reduce stress shielding and thigh pain by changing the transmission of stem load [17].

Tri-Lock BPS manages soft tissue laxity without affecting leg length by choosing different offset stems, including standard and high offset stems [18]. Femoral offset (FO) is defined by the distance between the center of the femoral head and a line bisecting the long axis of the femur [19]. Restoring the offset and leg length is crucial for optimal function and long-term outcomes following THA [20]. An appropriate offset is associated with a better soft-tissue tension, a better range of motion (ROM), and a lower dislocation rate [21–23]. Femoral stems comprise different offset versions, which effectively restore the biomechanical hip [24]. The high offset stem has been confirmed to decrease the risk of dislocation than the standard offset stem after THA [25]. However, high offset stems are subject to increase torsional loading about the long axis of the implant and increase the loosening rate in cemented high offset stems [26].

Although the high offset BPS was used widely, few studies explored the clinical and radiological results. Given that any design of an implant demands a careful follow-up, we conduct this specific study on outcomes of high offset Tri-Lock BPS in unilateral primary THA. The purpose of this study was to determine the clinical and radiological results of high offset Tri-Lock BPS in unilateral primary THA at a minimum follow-up of 3 years.

## Methods

### Inclusion and exclusion criteria

The inclusion criterion was as follows: (1) patients who underwent primary THAs in our institution without contraindication; (2) patients using high offset Tri-lock BPS (Depuy, Johnson & Johnson, Warsaw, IN, USA) in the THA surgery; (3) patients had enough radiological and clinical data; (4) patients had a contralateral native hip;

The exclusion criterion was as follows: (1) patients with one-stage bilateral THA; (2) patients who were diagnosed as dysplasia of the hip (DDH) Crowe type III or IV; (3) patients who had disqualified or incomplete radiological data; (4) patients who were lost to follow up; (5) patients had a contralateral hip with prosthesis or deformity.

### Study population

This retrospective study was approved by the clinical trials and biomedical ethics committee of West China Hospital and written informed consents were obtained from all the participants. From April 2017 to April 2018, 80 patients (87 hips) were identified who underwent cementless THAs with high offset Tri-lock BPS in our institution by five experienced senior surgeons. Of all the 80 patients, 7 patients (14 hips) were one-stage bilateral THAs, 6 patients (6 hips) had disqualified radiological data, 5 patients (5 hips) were lost to follow up, 6 patients (6 hips) had a contralateral hip with prosthesis or deformity, and 1 patient died for cholangiocarcinoma, which was unrelated to the THA surgery. Thus, 55 hips in 55 patients comprised the study population. Patients' demographic and characteristic data were collected from the electronic medical data (Table 1).

**Table 1:** Preoperative characteristics

Parameters	
Number of hips (patients)	55 (55)
Age (years)	49.8 (25–73)
Sex (female: male)	13 (23.6%): 42 (76.4%)
BMI (kg/m^2^)	23.8 (17.9–33.8)
Surgery side (left: right)	20:35
Main diagnosis (*n*, percentage)	
Primary OA	4 (7.3%)
DDH	10 (18.2%)
ONFH	29 (52.7%)
Femoral neck fracture	1 (1.8%)
OA secondary to childhood hip problems	2 (3.6%)
OA secondary to infection	7 (12.7%)
Post-traumatic osteoarthritis	2 (3.6%)
Dorr type (*n*, percentage)	
A	10 (18.2%)
B	43 (78.2%)
C	2 (3.6%)
Mean follow-up (months)	42.5 (36–48)

BMI, body mass index; OA, Osteoarthritis; DDH, dysplasia of the hip; ONFH, osteonecrosis of the femoral head

### Surgical technique

Preoperative templating in the standard anteroposterior (AP) pelvic radiograph was used to identify appropriate acetabular size and location. Appropriate femoral stem size and neck length were confirmed to fit the geometry of the femur and acquired proper hip offset (HO) and leg length discrepancy (LLD) then. Tri-lock BPS dual offset options provided surgeons with standard and high offset stems to restore femoral offset without affecting leg length. We only included patients who use high offset Tri-lock BPS during the THA in our study. All patients were treated with general anesthesia. Pinnacle acetabular cup systems (Depuy, Johnson & Johnson, Warsaw, IN, USA) were used in the surgeries, including 32/36 mm ceramic heads and appropriate liner. A polyethylene/ceramic liner accommodating a ceramic head was used in all the patients. All the surgeries were performed through the posterolateral (PL) approach or direct anterior approach (DAA). Conventional prophylactic intravenous antibiotics and thromboprophylaxis were used postoperatively. Furthermore, patients were encouraged to take active exercise in bed after surgery. On the second day postoperatively, partial weight training with the help of a walking aid was conducted after confirming the AP pelvic and lateral X-rays. We educated patients to walk with a walking stick two weeks postoperatively. They gradually change to full weight-bearing training four weeks postoperatively. Patients were followed up at two weeks, four weeks, three months, six months, 12 months, and annually postoperatively.

### Clinical assessment

Electronic medical records were evaluated to obtain all the in-hospital complications. Complications such as wound infection, periprosthetic infection (PJI), deep venous thrombosis (DVT), pulmonary embolism (PE), periprosthetic fracture (PFF), dislocation, and persistent thigh pain were recorded during the follow-up. Reoperation and revision for any reason were recorded during the follow-up period. Harris hip score (HHS), Western Ontario and McMaster Universities Osteoarthritis Index (WOMAC), Oxford Hip Score (OHS) were used to assess the clinical function of the patients preoperatively and at the latest follow-up. OHS, WOMAC and HHS were classic and common functional hip scores and widely used to report clinical outcomes following THA [27, 28].

### Radiographic measurement

**Fig. 1:**
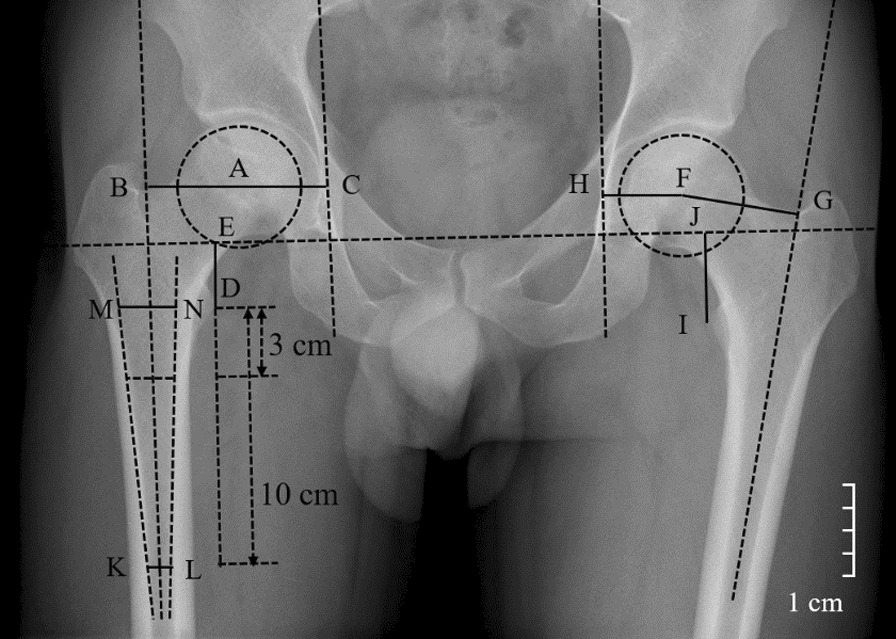
Radiographic measurement. The calcar width was measured as the middle level of the lesser trochanter (line MN). The canal width was measured as 10 cm below the lesser trochanter (line KL). The calcar-to-canal ratio was calculated as line KL divided by line MN, which was used for the Dorr classification. The femoral offset (FO) was measured as the vertical distance from the center of COR and the ipsilateral anatomical femoral axis (line AB and line FG). The acetabular offset (AO) was measured as the vertical distance from the COR and the vertical line passing through the ipsilateral teardrop (line AC and line FH). The hip offset (HO) was calculated as the sum of FO and AO (line AB + line AC and line FG + line FH). Leg length discrepancy (LLD) was calculated as the difference between the bilateral vertical line from the most prominent part of the trochanter to the transteardrop line (difference of line ED and line JI)

**Fig. 2:**
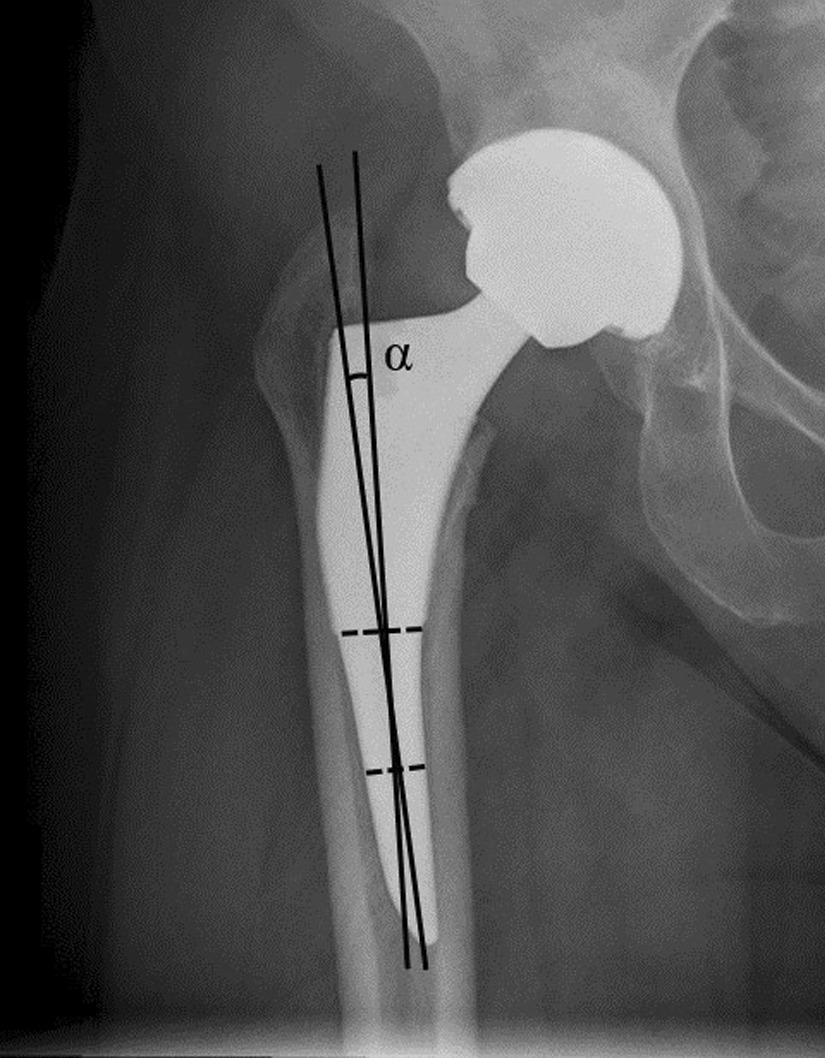
The stem alignment was defined as neutral (deviation from the axis of the femoral shaft within 5°), valgus (lateral deviation more than 5°), or varus (medial deviation more than 5°)

All the radiographs were collected and measured by two independent reviewers who were unrelated to the surgeries and blinded to the clinical outcomes. Another measurement was performed by the same two observers one week after the initial measurement, blinded to the previous results. Radiographic assessment was measured on the AP pelvic radiograph with the lower limb in 15° internal rotation using Syngo (Siemens Medical Solutions, Forchheim, Germany) preoperatively, second day postoperatively, and at the latest follow-up. The preoperative geometry of proximal femur was classified by the classification system of Dorr et al. (Fig. 1) [29]. The calcar-to-canal ratio was calculated by the calcar width (the middle level of the lesser trochanter) divided by the canal width (10 cm below the lesser trochanter) (Fig. 1) [30]. Therefore, the geometry of proximal femur was divided into type A (0–0.500), type B (0.501–0.750), and type C (0.751–1.000) according to the calcar-to-canal ratio [31]. Other radiographic parameters were measured and calculated, including FO, acetabular offset (AO), HO, HO difference, and LLD in the AP pelvic radiograph preoperatively and second day postoperatively (Fig. 1) [32]. The FO was measured as the vertical distance from the center of rotation of the femoral head (COR) and the ipsilateral anatomical femoral axis [32]. The AO was measured as the vertical distance from the COR and the vertical line passing through the ipsilateral teardrop [32]. The HO was calculated as the sum of FO and AO [32]. The HO difference was calculated as the difference between the bilateral HO. LLD was calculated as the difference between the bilateral vertical line from the most prominent part of the trochanter to the transteardrop line (TTL) [33]. Standard pelvic AP and lateral radiographs postoperatively and at the latest follow-up were used to evaluate for evidence of bone ingrowth, stem subsidence, stem alignment, radiolucent line around the stem, osteolysis, loosening, ectopic ossification, and femoral stress shielding [34]. The bone ingrowth was identified according to Engh et al. [35, 36]. The stem subsidence was identified if the femoral stem settled more than 3 mm between the immediate postoperative radiographs and those at the last follow-up [35]. The stem alignment was defined as neutral (deviation from the axis of the femoral shaft within 5°), valgus (lateral deviation more than 5°), or varus (medial deviation more than 5°) according to the previous study (Fig. 2) [37]. The radiolucent line was defined as regular, linear, lucent areas around the stem [11]. Osteolysis was identified as at least 5 mm irregularly shaped radiolucent at the bone-stem interface [36, 38]. Ectopic ossification was identified according to the Brooker classification [39]. Femoral stress shielding was identified according to a modification of the criteria defined by Engh and Bobyn and divided into four degrees [34].

### Statistical analysis

The data were analyzed using SPSS 26.0 (IBM, Armonk, USA). A two-tailed paired t-test was used to assess the differences between the FO, HO, HO difference, and LLD preoperatively and on the second day postoperatively. A two-tailed paired t-test was used to assess the HHS, WOMAC, OHS scores. Intraclass correlation coefficients (ICC) were used to measure intraobserver and interobserver reliability with a two-way random model for absolute agreement. P-value < 0.05 was defined as statistical significance.

## Results

### General information

The mean age of the study population was 49.8 years (range 25–73). Our study included 13 females (23.6%) and 42 males (76.4%). Patients' mean body mass index (BMI) was 23.8 kg/m^2^(range 17.9–33.8). Twenty patients had left side surgeries, while 35 patients had right. The most common diagnosis was osteonecrosis of the femoral head (ONFH), which was found in 29 (52.7%) patients. According to the Dorr classification, ten patients (18.2%) were classified as Dorr A, 43 patients (78.2%) as Dorr B, and two patients (3.6%) as Dorr C. And the mean follow-up was 42.5 months (range 36–48) (Table 1).

Thirty patients (54.5%) underwent THA through a PL approach, while 25 patients (45.5%) through a DAA approach. The mean stem size was 3.3# (range 0#-8#). Nineteen patients (34.5%) received 32 mm Ceramic femoral heads, while 36 patients (65.5%) received 36 mm ones. The bearing surface was ceramic-on-ceramic in 50 patients (90.9%) and ceramic-on-polyethylene in 5 patients (9.1%). Fifty-three patients (96.4%) had a neutral stem alignment, one patient (1.8%) had valgus, and one (1.8%) had varus (Table 2).

**Table 2:** Surgical and prosthesis data

Parameters	
Surgical approach (*n*, percentage)	
PL	30 (54.5%)
DAA	25 (45.5%)
Stem size	3.3 (0–8)
Ceramic femoral head size (*n*, percentage)	
32 mm	19 (34.5%)
36 mm	36 (65.5%)
Bearing surface	
Ceramic-on-ceramic	50 (90.9%)
Ceramic-on-polyethylene	5 (9.1%)
Stem alignment	
Neutral	53 (96.4%)
Valgus	1 (1.8%)
Varus	1 (1.8%)

PL, posterolateral approach; DAA, direct anterior approach

### Clinical results

During the follow-up, no patients reported any complications, including wound infection, PJI, DVT, PE, PFF, dislocation, nor persistent thigh pain. Furthermore, there were no patients who encounter any revision surgery or reoperation. The survival rate of high offset Tri-Lock BPS at the latest follow-up was 100%.

The HHS showed a significant improvement from 48.13 ± 9.66 preoperatively to 96.84 ± 5.60 at the latest follow-up (*p* < 0.01). At the latest follow-up, all the patients (100%) had excellent or good function results on HHS**.** The WOMAC total score decreased from 50.04 ± 9.40 preoperatively to 3.27 ± 3.36 at the latest follow-up (*p* < 0.01). Besides, the OHS decreased from 36.15 ± 8.80 preoperatively to 15.33 ± 3.12 at the latest follow-up (*p* < 0.01) (Table 3).

**Table 3:** Clinical results

Parameters	Preoperative	At latest follow-up	*p* value
HHS	48.13 ± 9.66	96.84 ± 5.60	< 0.01
Excellent (90–100)	0	50	
Good (80–89)	0	5	
Fair (70–79)	0	0	
Poor (< 70)	55	0	
WOMAC total score	50.04 ± 9.40	3.27 ± 3.36	< 0.01
Pain	8.87 ± 2.42	1.04 ± 1.28	< 0.01
Stiffness	2.96 ± 1.20	0.67 ± 0.70	< 0.01
Function	38.20 ± 9.87	1.56 ± 1.76	< 0.01
OHS score	36.15 ± 8.80	15.33 ± 3.12	< 0.01

HHS, Harris hip score; WOMAC, Western Ontario and McMaster Universities Osteoarthritis Index; OHS, Oxford Hip Score

### Radiographic results

The ICCs for intraobserver and interobserver agreement among all the radiographic parameters were excellent (> 0.8). The mean FO significantly improved from 43.2 ± 9.1 mm to 48.2 ± 7.4 mm (*p* < 0.01). The HO improved from 81.0 ± 8.6 mm to 83.4 ± 8.5 mm (*p* < 0.01). The HO difference between legs decreased from 2.8 ± 7.0 mm preoperatively to 0.4 ± 5.7 mm postoperatively (*p* < 0.01). Besides, the LLD decreased from 7.4 ± 8.4 mm to 4.3 ± 3.3 mm (*p* < 0.01). On the second day postoperatively, 53 patients (96.4%) limited LLD within 10 mm, and all the patients limited LLD within 20 mm (Table 4).

**Table 4:** Radiographic results

Parameters	Pre-op	2nd day post-op	*p* value
FO (mm)	43.2 ± 9.1	48.2 ± 7.4	< 0.01
HO (mm)	81.0 ± 8.6	83.4 ± 8.5	< 0.01
HO difference (mm)	2.8 ± 7.0	0.4 ± 5.7	< 0.01
LLD (mm)	7.4 ± 8.4	4.3 ± 3.3	< 0.01
0–10 mm	41 (74.5%)	53 (96.4%)	
10–20 mm	10 (18.2%)	2 (3.6%)	
> 20 mm	4 (7.3%)	0 (0.0%)	

FO, femoral offset; HO, hip offset; LLD, leg length discrepancy

**Fig. 3:**
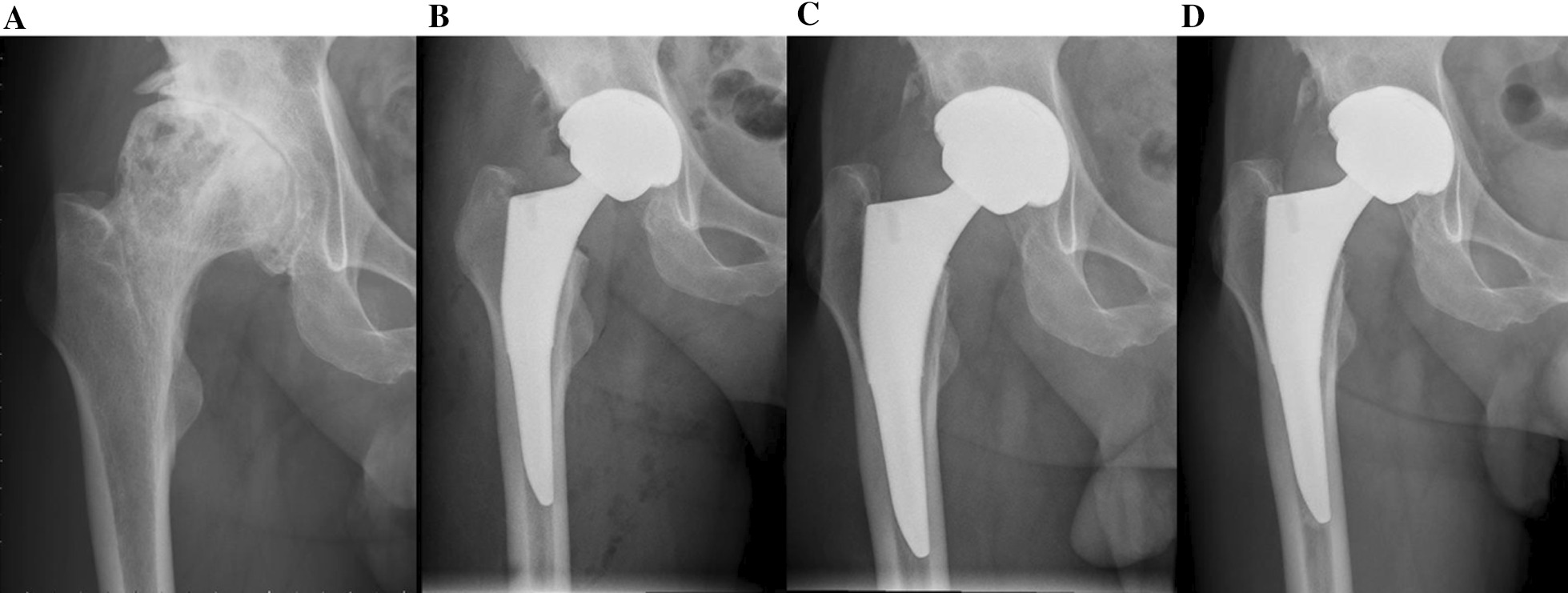
Anteroposterior pelvic radiograph of a 57-year-old male who was diagnosed as OA secondary to infection. The images show the progression of stress shielding at preoperative (**a**), second day postoperatively (**b**), 1 year (**c**), and 4 years post-operatively (**d**)

The radiographic evaluation confirmed bone ingrowth in all the patients. Fifty-three patients (96.4%) had a neutral stem alignment. Only one patient (1.8%) had a valgus stem, and another (1.8%) had a varus stem. Those two patients also achieved good clinical and radiographic outcomes at the latest follow-up. There were no radiographic signs of stem subsidence, radiolucent line, osteolysis, loosening, or ectopic ossification. Stress shielding was observed in 55 hips (100%). Among them, 32 patients (58.2%) were classified in grade 1 and 23 patients (41.8%) were classified in grade 2 (Fig. 3). No patients detected severe stress shielding (grade 3 or 4) in our study.

## Discussion

This study mainly evaluated the clinical and radiological results of high offset Tri-Lock BPS in unilateral primary THA at a minimum follow-up of 3 years. No patients reported any complications related to the stem during hospitalization nor the follow-up period. At a mean follow-up of 42.5 months, The HHS, WOMAC, and OHS scores showed a significant improvement. No patients reported thigh pain. The survival rate of high offset Tri-Lock BPS at a minimum follow-up of 3 years was 100% as no patients suffered any revision nor radiographic loosening. Besides, the high offset Tri-Lock BPS significantly improved the FO and HO postoperatively. HO difference and LLD were balanced postoperatively. At the latest follow-up, no signs of stem subsidence, radiolucent line, osteolysis, loosening, or ectopic ossification were observed.

Some previous studies found that the Tri-Lock BPS offers excellent postoperative function at a mid to long follow-up, which were similar to our results [15, 18, 40]. A recent study found no comparable significance between Tri-Lock BPS and conventional long stem in terms of HHS, VAS, and WOMAC [11]. 96.4% of patients had a neutral stem alignment postoperatively in our study. Patients with valgus or varus stem alignment did not get any inferior clinical or radiological results, which was consistent with the previous study [18]. Ulive et al. confirmed that varus and valgus in terms of stem alignment did not undermine implants' survival and clinical and patient-reported outcomes [18].

No patients reported any complications, including dislocation during the follow-up period in our study. The other two studies reported postoperative dislocation with Tri-Lock BPS stem during the follow-up period [11, 18]. However, they failed to report if high offset stems were used in the dislocation patients. In a retrospective study involving more than ten hundred patients, 49% utilized high offset stems. There were 51 (0.41%) patients who occurred dislocations postoperatively. Of those patients who occurred hip dislocations, only 2 (4%) patients utilized standard offset stems [25]. The use of a high offset stem may decrease the risk of dislocation.

In our study, no patient experienced persistent thigh pain. This low incidence of thigh pain was consistent with previous studies [15, 18]. In the study of Guo et al., the rate of thigh pain was 0% at a mean follow-up of 48 months [11]. Two meta-analyses emphasized that short stems decreased the incidence of thigh pain [41, 42]. This superiority of short stem might be related to the reduced proximal stress shielding and the development of excellent mechanical transmission [43].

In this study, the specific stem was stable and showed no signs of stem subsidence. Some previous literature reported the outcomes of Tri-Lock BPS regardless of standard or high-offset version stems [11, 15, 18, 40]. Albers et al. [15] reported a 99.2% stem survival rate of Tri-Lock BPS at a minimum 4-year follow-up. Ulivi et al. [18] reported that the survival rate of Tri-Lock BPS was 99% at a mean follow-up of 5.7 years. One patient received revision surgery for hip dislocation [18]. Zhen et al. [40] investigated patients who use Tri-Lock BPS in Dorr type C femoral bone. At a mean follow-up of 5.5 years, no signs of stem subsidence were observed. The high survival rate may be related to the unique design and the roughened porous coating [15]. Guo et al. found that one patient required revision because of recurrent dislocation and no occurrences of prosthesis subsidence in the cohort of 104 hips at a mean follow-up of 2 years [11]. The proximal porous coating maintains mechanical integrity under shear, compression, torsion, and tension force [16]. A shorter length and narrow distal segment allowed a better proximal stress transfer and avoided distal stress overload [18]. Extended osteotomy may be needed for the femoral stem removal during potential future revision THA [44]. However, no revision surgery had been detected in our cohort. We need to notice the potential risk of extended osteotomy in future revision THA.

No patients detected severe (grade 3 or 4) stress shielding in our study. Our results were consistent with previous studies. Zhen et al. [40] found that no patients exhibited severe stress shielding at a mean follow-up of 5.5 years. In the study of Guo et al., 9% of patients detected grade 3 stress shielding at a mean follow-up of 48 months [11]. Tri-Lock BPS stem can stock the proximal femoral bone from loss and get the primary stability by a metaphyseal fixation. The unique design reduced the incidence of the distal medullary cavity being invaded and stress shielding [41].

HO and leg length reconstruction postoperatively is crucial for an additive effect on clinical outcomes [20]. Restoration of leg length and offset correlate reduced trochanteric pain syndrome postoperatively [45]. A postoperative unbalanced offset between legs was associated with hip abductor muscle weakness and may increase gait asymmetry in the sagittal plane [21]. Leg length discrepancy is one of the most common causes of litigation following THA [46]. The use of a high offset stem offered better restoration of the offset [47]. Although the high offset stem was used widely, few studies have quantified the improvement of offset and LLD by the specific stem design. We found that the FO and HO were significantly improved from preoperative to postoperative in our study. HO difference and LLD between legs decreased significantly and achieved balance postoperatively. The use of high offset stems helped surgeons achieving excellent HO and LLD in our cohort. Incavo et al. found 85% of patients had no clinical leg length discrepancy postoperatively using a high offset stem in primary THA [48]. However, they did not provide a quantitative measurement of the improvement on offset and LLD. Yao et al. [49] revealed Tri-Lock stem restores the offset and LLD. But they did not provide the proportion of dual offset stems, including standard and high offset stems [49]. A meta-analysis including 320 patients reported no significant differences in the FO and LLD after primary THA using short stem versus conventional stem [42]. This was the first study that has quantified the improvement of offset and LLD using a high offset Tri-Lock stem. In order to restore offset, using a standard offset stem may lengthen the neck and thus lead to leg length discrepancy for patients with native high offset. The Tri-Lock BPS is available with standard and high offset versions for all the stem sizes. The high offset stem provides direct lateralization, increasing offset without sacrificing leg length. The excellent functional outcomes of the patients in our study might be attributed to the restoration of offset and LLD by high offset Tri-Lock stem.

The strengths of this study were its completeness of the clinical and radiological results of high offset Tri-Lock BPS at a minimum follow-up of 3 years. Moreover, we have quantified the improvement of offset and LLD firstly by using this specific stem.

There are also some limitations to our study. First, the sample size of this study was relatively small and lacked a control group. Second, this was a retrospective study and the risk of selection bias cannot be avoided. Third, all the patients were followed for not more than 4 years. Further studies are required to detect long-term clinical and radiological results of high offset Tri-Lock BPS.

## Conclusions

The high offset Tri-Lock BPS demonstrated excellent clinical and radiographic outcomes at a minimum follow-up of 3 years. There were no radiographic signs of stem subsidence, radiolucent line, osteolysis, loosening, or ectopic ossification. HO difference and LLD between legs decreased significantly and achieved balance postoperatively. Extended follow-up is required to assess the durability of this stem.

## Data Availability

The authors confirm that the data supporting the findings of this study are available within the article.
